# The Role of Emphysema on Postoperative Prognosis in Early-Stage Nonsmall Cell Lung Cancer

**DOI:** 10.1245/s10434-024-15126-x

**Published:** 2024-05-13

**Authors:** Masayuki Ishida, Takahiro Mimae, Atsushi Kamigaichi, Nobutaka Kawamoto, Norifumi Tsubokawa, Yoshihiro Miyata, Morihito Okada

**Affiliations:** Department of Surgical Oncology, Hiroshima University, Hiroshima, Japan

**Keywords:** Emphysema, Nonsmall cell lung cancer, Surgery, Prognosis, Complications

## Abstract

**Background:**

Emphysema is generally considered a poor prognostic factor for patients with nonsmall cell lung cancer; however, whether the poor prognosis is due to highly malignant tumors or emphysema itself remains unclear. This study was designed to determine the prognostic value of emphysema in patients with early-stage nonsmall cell lung cancer.

**Methods:**

A total of 721 patients with clinical stage IA nonsmall cell lung cancer who underwent complete resection between April 2007 and December 2018 were retrospectively analyzed regarding clinicopathological findings and prognosis related to emphysema.

**Results:**

The emphysematous and normal lung groups comprised 197 and 524 patients, respectively. Compared with the normal lung group, lymphatic invasion (23.9% vs. 14.1%,* P* = 0.003), vascular invasion (37.6% vs. 17.2%, *P* < 0.001), and pleural invasion (18.8% vs. 10.9%, *P* = 0.006) were observed more frequently in the emphysema group. Additionally, the 5-year overall survival rate was lower (77.1% vs. 91.4%, *P* < 0.001), and the cumulative incidence of other causes of death was higher in the emphysema group (14.0% vs. 3.50%, *P *< 0.001). Multivariable Cox regression analysis of overall survival revealed that emphysema (vs. normal lung, hazard ratio 2.02, *P* = 0.0052), age > 70 years (vs. < 70 years, hazard ratio 4.03, *P* < 0.001), and SUVmax > 1.8 (vs. ≤ 1.8, hazard ratio 2.20, *P* = 0.0043) were independent prognostic factors.

**Conclusions:**

Early-stage nonsmall cell lung cancer with emphysema has a tendency for the development of highly malignant tumors. Additionally, emphysema itself may have an impact on poor prognosis.

**Supplementary Information:**

The online version contains supplementary material available at 10.1245/s10434-024-15126-x.

Pulmonary emphysema mainly results from the destruction of the alveolar wall because of smoking and is characterized by abnormal and permanent enlargement of airspaces distal to the terminal bronchioles.^1^ Although adhesion molecules, such as cell adhesion molecule, have been reported to contribute to the etiology of emphysema, the detailed pathogenesis of pulmonary emphysema is not fully understood.^1,2^

Chronic obstructive pulmonary disease (COPD), a common respiratory disease, is associated with high morbidity and mortality worldwide. Emphysema is a classic subtype of COPD.^3^ Pulmonary emphysema often coexists with primary lung cancer, and one report indicated that emphysema was present in 40%–70% of cases of primary lung cancer.^4^ Furthermore, lung cancer arising from emphysema often is highly malignant. In one report, squamous cell carcinoma and small cell carcinoma were more common in patients with emphysematous lungs than in patients with normal lungs, while adenocarcinoma was less common.^5^ Moreover, another report found that patients with lung cancer and COPD have worse overall survival (OS) than those with normal lungs.^6^ In addition, poor prognosis has been reported in cases of primary lung cancer with emphysema or combined pulmonary fibrosis and emphysema detected using computed tomography (CT).^7,8^ Whether the poor prognosis of patients with lung cancer and emphysema is the result of the emphysema itself or the high-grade malignancy of lung cancer, which is observed more frequently in emphysematous lungs, remains unknown.

In the present study, we attempted to elucidate the role of emphysema in the prognosis of patients with nonsmall cell lung cancer (NSCLC) in terms of tumor malignancy and the presence of emphysema itself. To minimize the complexity of advanced NSCLC in terms of prognosis, the early stage (i.e., clinical stage IA NSCLC) was selected for this study. The results of the present study may provide useful information for physicians involved in lung cancer practice in surgical treatment and postoperative follow-up.

## Methods

### Study Population

This retrospective study included 721 patients who underwent complete resection at Hiroshima University for clinical stage IA NSCLC tumors between April 2007 and December 2018. The institutional review boards at the participating institutions approved this retrospective review of a prospective database and waived the requirement for informed consent from individual patients (E2018-1216-02, November 30, 2022). Patients were divided into two groups: 524 with normal lungs (normal lung group) and 197 with emphysematous lungs (emphysema group). Patients with radiological patterns of interstitial pneumonia (IP) or those who received preoperative induction therapy were excluded (Supplementary Fig. 1).

Clinicopathological findings, including age, sex, smoking status, performance status, pulmonary function test, imaging findings, tumor markers, histology, surgical procedure, and postoperative complications, were evaluated. Patient comorbidities were scored by using the Charlson comorbidity index (CCI) based on database records.^9^ Clinical stages were determined according to the 8th edition of the TNM classification.^10^ Endobronchial ultrasonography and mediastinoscopy were not performed routinely. Lymph node metastasis was determined to be negative when the short axis of the mediastinal or hilar lymph nodes was <1 cm on high-resolution computed tomography (HRCT) and when 18F-fluorodeoxyglucose (FDG) did not accumulate in these nodes on FDG-positron emission tomography (PET) images.

### Evaluation of Emphysema

Pulmonary emphysema is characterized by an abnormal and permanent enlargement of the peripheral air space, which is normally thinner than the terminal bronchus, and destruction of the alveolar wall. CT is useful for identifying pulmonary emphysema and assessing its severity.^11,12^ In the present study, emphysema was defined as a Goddard score ≥ 1, and the severity was classified as mild (score, 1–7), moderate (score, 8–15), or severe (score, 16–24). Briefly, the Goddard score is used to assess the severity of emphysema in the lung field during CT imaging.^13^ The presence of emphysema was prospectively determined by a preoperative CT review by the tumor board, which comprised surgical oncologists, medical oncologists, pulmonologists, radiologists, and pathologists.

### Follow-Up Evaluation

All patients received follow-up from the day of surgery. In general, patients were followed up at 3–6-month intervals for 2 years, then 6–12-month intervals for the next 3 years, and annually as required. Follow-up evaluations included physical examination and routine laboratory tests, including tumor markers, chest x-rays, and chest and abdominal CT. FDG-PET and brain magnetic resonance imaging were requested based on the clinical symptoms. Postoperative complications were defined according to the Clavien–Dindo classification.^14^ When a patient died during follow-up, the death was defined as either a lung cancer-related death or as any other cause of death.

### Statistical Analysis

Data are presented as numbers (%) or medians unless otherwise stated. Continuous variables were analyzed using the Mann–Whitney *U* test, and categorical variables were assessed using the chi-square test or Fisher’s exact test. Overall survival and recurrence-free survival (RFS) were calculated by using the Kaplan–Meier method. Overall survival was defined as the time interval from the date of surgery to the time of death as a result of any cause or the last follow-up visit. Recurrence-free survival was defined as the time interval from the date of surgery to the time of recurrence or death, whichever occurred first. The risk of death was estimated using a cumulative incidence function that considered lung cancer-related deaths and other causes of death as competing events. Differences in the cumulative incidence between the groups were assessed by using the Gray methods.^15^ Emphysematous lungs (vs. normal lungs), age > 70 years (vs. age < 70 years), male sex (vs. female sex), pure solid nodule (vs. nodule containing GGO), solid size > 20 mm (vs. solid size ≤ 20 mm), maximum standardized uptake value (SUVmax) of > 1.8 (vs. SUVmax ≤ 1.8), and CCI score ≥ 3 (vs. CCI score < 3) were selected as variables in univariate and multivariate analyses. These variables were selected because the prognosis after pulmonary resection is generally poor in elderly patients, male patients, and patients who have tumors without GGO components, with large invasive diameters, with high FDG accumulations, and with high CCI scores.^16–21^ Propensity scores were calculated by using a logistic regression model based on preoperative characteristics that included age > 70 years of (vs. age < 70 years), male sex (vs. female sex), pure solid nodule (vs. nodule containing GGO), solid size > 20 mm (vs. solid size ≤ 20 mm), SUVmax > 1.8 (vs. SUVmax ≤ 1.8), and CCI score ≥ 3 (vs. CCI score < 3). In addition, stratified propensity scores were included as covariates in multivariable Cox regression analyses of OS. Statistical significance was set at *P* < 0.05. All statistical analyses were performed by using EZR (Saitama Medical Center, Jichi Medical University, Saitama, Japan), a graphical user interface for R (The R Foundation for Statistical Computing, Vienna, Austria, version 4.1.2).^22^ More precisely, EZR is a modified version of the R commander designed to add statistical functions frequently used in biostatistics.

## Results

### Patients and Tumor Characteristics with or without Emphysema

Tables 1 and 2 summarize the characteristics of the 721 patients included in this study. In the normal lung and emphysema groups, the median ages were 68 years and 69 years, 234 (44.7%) and 182 (92.4%) of the patients were men, and the median smoking histories were 0 and 40 pack-years, respectively. The CCI score was significantly higher in the emphysema group (normal lung vs. emphysema, 0 vs. 1, *P* < 0.001). The overall tumor diameter was similar in both groups (normal lung vs. emphysema, 19.0 mm vs. 19.6 mm, *P* = 0.95). However, compared with those in the normal group, in the emphysema group, the ground-glass opacity (GGO) rate was lower (20.0% vs. 0.00%), the solid diameter of tumors was larger (13.5 mm vs. 16.0 mm), and the maximum standardized uptake value (SUVmax) in tumors was higher (1.6 vs. 2.7) (all *P* < 0.001). Lobectomy (279 [53.2%] vs. 96 [48.7%]), segmentectomy (173 [33.0%] vs. 67 [34.0%]), and wedge resection (72 [13.7%] vs. 34 [17.3%]) were performed in each group.

**Table 1:** Clinical characteristics of patients

Variables	Overall (*n* = 721)	Normal lungs (*n* = 524)	Emphysema (*n* = 197)	*P*
Age (year)	68 (62–74)	68 (62–74)	69 (64–75)	0.017*
Sex				
Male	416 (57.7 %)	234 (44.7 %)	182 (92.4 %)	< 0.001*
Pack year	10 (0–46)	0 (0–25)	40 (50–69)	< 0.001*
PS				
0	305 (42.3 %)	208 (39.7%)	97 (49.2%)	< 0.001*
1	30 (4.2%)	11 (2.1%)	19 (9.6%)	
2	0 (0.0%)	0 (0.0%)	0 (0.0%)	
3	1 (0.1%)	1 (0.2%)	0 (0.0%)	
CCI	0 (0–1)	0 (0–1)	1 (0–2)	< 0.001*
%VC (%)	99.7 (88.5–109.7)	100.7 (90.1–110.5)	94.8 (83.9–107.0)	< 0.001*
VC (L)	3.03 (2.50–3.65)	2.91 (2.42–3.52)	3.31 (2.79–3.92)	< 0.001*
FEV1.0 (L)	2.21 (1.82–2.66)	2.23 (1.85–2.65)	2.15 (1.64–2.68)	0.068
FEV1.0% (%)	75.0 (68.3–80.3)	76.7 (71.9–81.3)	67.5 (58.0–75.1)	< 0.001*
Whole tumor size (mm)	19.0 (14.0–25.5)	19.0 (14.0–25.0)	19.6 (14.7–26.0)	0.95
GGO rate (%)	0.00 (10.0–47.8)	20.0 (0.00–50.0)	0.00 (0.00–28.0)	< 0.001*
Solid tumor size (mm)	14.4 (9.00–21.0)	13.5 (8.30–20.0)	16.0 (12.0–22.7)	< 0.001*
cT-factor				
T1mi	82 (11.4%)	69 (13.2%)	13 (6.6%)	< 0.001*
T1a	137 (19.0%)	113 (21.6%)	24 (12.2%)	
T1b	314 (43.6%)	220 (42.0%)	94 (47.7%)	
T1c	188 (26.1%)	122 (23.3%)	66 (33.5%)	
CEA (ng/mL)	2.5 (1.8–4.2)	2.3 (1.6–3.7)	3.3 (2.3–5.8)	0.002*
CYFRA (ng/mL)	1.9 (1.4–2.6)	1.8 (1.4–2.4)	2.1 (1.6–2.8)	0.021*
SUVmax	1.8 (1.1–3.7)	1.6 (0.95–3.0)	2.7 (1.5–5.6)	< 0.001*

Variables with statistically significant differences are marked with *

Values are shown as medians (interquartile range) or as n (%)

*PS* performance status; *CCI* Charlson Comorbidity Index; *VC* vital capacity; *FEV1.0* forced expiratory volume in 1 s; *GGO* ground glass capacity; *SUVmax* maximum standardized uptake value

**Table 2:** Postoperative characteristics of patients

Variables		Overall (*n* = 721)	Normal lungs (*n* = 524)	Emphysema (*n* = 197)	*P*
Procedure	Lobectomy	375 (52.0%)	279 (53.2%)	96 (48.7%)	0.40
	Segmentectomy	240 (33.3%)	173 (33.0%)	67 (34.0%)	
	Wedge resection	106 (14.7%)	72 (13.7%)	34 (17.3%)	
Histology	Adenocarcinoma	604 (83.8%)	478 (91.2%)	126 (64.0%)	< 0.001*
	Squamous cell carcinoma	69 (9.6%)	24 (4.6%)	45 (22.8%)	
	Large cell carcinoma	20 (2.8%)	8 (1.5%)	12 (6.1%)	
	Pleomorphic carcinoma	7 (1.0%)	2 (0.4%)	5 (2.5%)	
	others	21 (2.9%)	12 (2.3%)	9 (4.6%)	
LY	1	121 (16.8%)	74 (14.1%)	47 (23.9%)	0.003*
V	1	164 (22.7%)	90 (17.2%)	74 (37.6%)	< 0.001*
PL	1	94 (13.0%)	57 (10.9%)	37 (18.8%)	0.006*
PM	1	16 (2.2%)	11 (2.1%)	5 (2.5%)	0.78
pN	1,2	44 (6.1%)	32 (6.1%)	12 (6.1%)	1.0
Recurrence		79 (11.0%)	46 (8.8%)	33 (16.8%)	0.003*

Variables with statistically significant differences are marked with *

Values are shown as medians (interquartile range) or as n (%)

*LY* lymphatic invasion; *V* vascular invasion; *PL* pleural invasion; *PM* pulmonary metastasis

For histological findings, while adenocarcinoma was the predominant histological type in the normal lung group, squamous cell carcinoma, large cell carcinoma, and pleomorphic carcinoma were significantly more common in the emphysema group (*P* < 0.001). In addition, significantly higher positive rates of lymphatic invasion (LY), vascular invasion (V), and pleural invasion (PL) were observed in the emphysema group (normal lung vs. emphysema, LY: 14.1% vs. 23.9%, *P* = 0.003; V: 17.2% vs. 37.6%, *P* < 0.001; PL: 10.9% vs. 18.8%, *P* = 0.006). The subtypes of adenocarcinoma were classified as follows: adenocarcinoma in situ, minimally invasive adenocarcinoma, and lepidic adenocarcinoma as low grade; papillary adenocarcinoma and acinar adenocarcinoma as moderate grade; solid adenocarcinoma and micropapillary adenocarcinoma as high grade; and invasive mucinous adenocarcinoma and others as others. With this classification, the high grade subtype was more common in the emphysema group (Supplementary Table 1; low grade: 37.2% vs. 23.0%, moderate grade: 57.3% vs. 64.3%, high grade: 4.80% vs. 11.1%, others: 0.60% vs. 1.6%, *P* = 0.018).

### Postoperative Complications with or without Emphysema

Table 3 shows the postoperative complications according to the Clavien-Dindo classification. Respiratory complications of Grade 2 or higher were significantly more frequent in the emphysema group (67 [12.8%] vs. 46 [23.4%], *P* < 0.001). Regarding respiratory complications, pneumonia and pulmonary fistulae were more frequently observed in the emphysema group.

**Table 3:** Postoperative complications (Clavien-Dindo classification ≥ 2)

	Overall (*n* = 721)	Normal lungs (*n* = 524)	Emphysema (*n* = 197)	*P*
All postoperative complications				
Respiratory	113 (15.7%)	67 (12.8%)	46 (23.4%)	< 0.001*
Cardiovascular	19 (3.6%)	12 (2.3%)	7 (3.6%)	0.43
CNS	6 (0.8%)	3 (0.6%)	3 (1.5%)	0.35
Gastrointestinal	12 (1.6%)	6 (1.1%)	6 (3.0%)	0.099
Others	36 (5.0%)	27 (5.2%)	9 (4.6%)	0.85
Respiratory postoperative complications				
BPF	4 (0.6%)	3 (0.6%)	1 (0.5%)	1.0
Chylothorax	4 (0.6%)	4 (0.8%)	0 (0.0%)	0.58
Empyema	4 (0.6%)	3 (0.6%)	1 (0.5%)	1.0
Pneumonia	17 (2.4%)	5 (1.0%)	12 (6.1%)	< 0.001*
Pulmonary fistula	76 (10.5%)	48 (9.2%)	28 (14.2%)	0.057
Sputum	4 (0.6%)	2 (0.4%)	2 (1.0%)	0.30

Variables with statistically significant differences are marked with *

*BPF* bronchopleural fistula; *CNS* central nervous system; *Sputum* cases requiring tracheal puncture or bronchoscopic expectoration because of sputum obstruction

### Prognosis of Patients with or without Emphysema

**Fig. 1:**
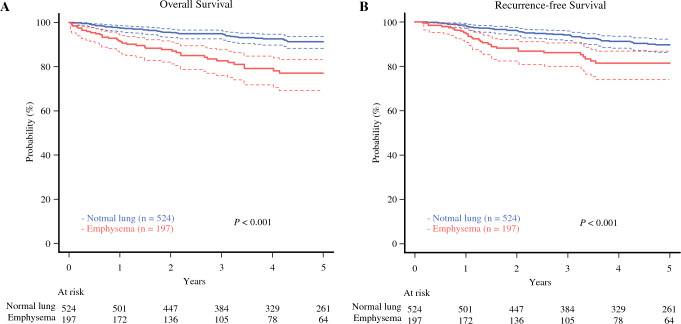
Comparison of overall survival and recurrence free survival in patients with normal or emphysematous lungs. **A** Five-year OS rates for patients with normal or emphysematous lungs (91.4% [95% CI 88.3–93.7%] vs. 77.1% [95% CI 69.2–83.1%], *P* < 0.01) and **B** RFS rates for patients with normal or emphysematous lungs (89.7% [95% CI 86.3%–92.3%] vs. 81.4% [95% CI 74.1–86.9%], *P* < .01), respectively. All *P* values were determined by log rank tests. *CI* confidence interval; *OS* overall survival; *RFS* recurrence-free survival

Figure 1 shows the OS and RFS curves for all patients. The 5-year OS and RFS rates were significantly worse in the emphysema group than those in the normal lung group (normal lung vs. emphysema, OS: 91.4% [95% confidential interval [CI] 88.3–93.7%] vs. 77.1% [95% CI 69.2–83.1%], *P* < 0.001; RFS: 89.7% [95% CI 86.3–92.3%] vs. 81.4% [95% CI 74.1–86.9%], *P* < 0.001). Multivariable analysis for OS revealed that emphysematous lungs (vs. normal lungs, hazard ratio [HR] 2.02, 95% CI 1.23–3.32, *P* = 0.0052), age >70 years (vs. age <70 years, HR 4.03, 95% CI 2.52–6.47, *P* < 0.001), and SUVmax >1.8 (vs. SUVmax ≤1.8, HR 2.20, 95% CI 1.28–3.79, *P* = 0.0043) were independent prognostic factors (Table 4). Moreover, an additional analysis with pathologic malignancy (the presence of LY, V, PL, PM, or lymph node metastases) and pathologic size as variables, instead of pure solid nodules and solid size on CT, showed that emphysema remained an independent prognostic factor (Supplementary Table 2). To minimize selection bias caused by imbalances of clinical characteristics, we performed propensity score matching. The multivariable Cox regression analysis, including propensity score as a variable, also revealed that emphysema was an independent prognostic factor (Supplementary Table 3).

**Table 4:** Univariable and multivariable Cox regression analyses for overall survival using clinical variables

Variables	Status	Univariable analysis		Multivariable analysis	
		HR (95% CI)	*P*	HR (95% CI)	*P*
Emphysema	Yes versus no	3.05 (2.00–4.64)	< 0.001*	2.02 (1.23–3.32)	0.0052*
Age (year)	≥ 70 versus < 70	3.40 (2.16–5.34)	< 0.001*	4.03 (2.52–6.47)	< 0.001*
Sex	Male versus female	2.28 (1.42–3.67)	< 0.001*	1.74 (1.00–3.02)	0.051
GGO status	Pure solid versus with GGO	2.51 (1.63–3.87)	< 0.001*	1.43 (0.86–2.37)	0.16
Solid size (mm)	> 20 versus ≤ 20	2.03 (1.32–3.12)	0.0013*	1.25 (0.78–2.01)	0.35
SUVmax	> 1.8 versus ≤ 1.8	3.08 (1.94–4.87)	< 0.001*	2.20 (1.28–3.79)	0.0043*
CCI	≥ 3 versus < 3	2.36 (1.28–4.36)	0.0062*	1.08 (0.57–2.06)	0.81

Variables with statistically significant differences are marked with *

*HR* hazard ratio; *CI* confidence interval; *GGO* ground glass capacity; *SUVmax* maximum standardized uptake value; *CCI* Charlson Comorbidity Index

**Fig. 2:**
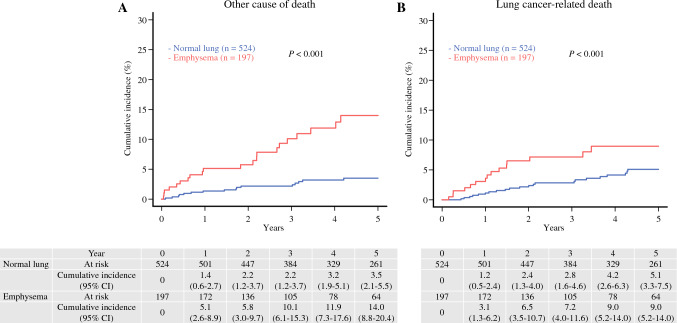
Comparison of cumulative incidences of other causes of death or lung cancer deaths in patients with normal or emphysematous lungs. **A** Cumulative incidence of other causes of death for patients with normal or emphysematous lungs (3.5% [95% CI 2.1–5.5%] vs. 14.0% [95% CI 8.8–20.4%], *P* < 0.01) and **B** lung cancer deaths for patients with normal or emphysematous lungs (5.1% [95% CI 3.3–7.5%] vs. 9.0% [95% CI 5.2%–14.0%], *P* = .017), respectively. All *P* values were determined by log-rank tests. Cumulative incidences are shown as percentages. *CI* confidence interval; *OS* overall survival; *RFS* recurrence-free survival

**Fig. 3:**
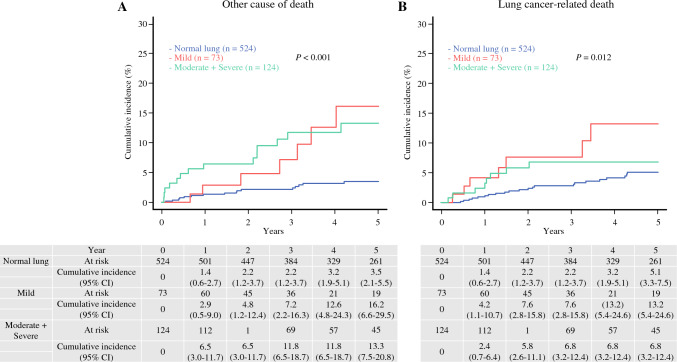
Comparison of cumulative incidences of other causes of death or lung cancer deaths in patients with normal lungs, mild emphysema, or moderate + severe emphysema. The emphysema group was divided into two subgroups: “mild”—a mild emphysematous group, and “moderate + severe”—a moderate-to-severe emphysematous group. **A** Cumulative incidences of other causes of death for patients with normal lungs, mild emphysema, or severe emphysema (3.5% [95% CI 2.1–5.5%] vs. 16.2% [95% CI 6.6–29.5%] vs. 13.3% [95% CI 7.5–20.8%], *P* < 0.01), and **B** lung cancer deaths for patients with normal lungs, mild emphysema, or severe emphysema (5.1% [95% CI 3.3%–7.5%] vs. 13.2% [95% CI 5.4–24.6%] vs. 6.8% [95% CI 3.2–12.4%], *P* = 0.012), respectively. All *P* values were determined by log-rank tests. *CI* confidence interval

Comparing the causes of death between the normal lung and emphysema groups, the cumulative incidence of other causes of death (normal lung vs. emphysema, 3.50% [95% CI 2.10–5.50%] vs. 14.0% [95% CI 8.80–20.4%], *P* < 0.001) and lung cancer-related deaths (normal lung vs. emphysema, 5.10% [95% CI 3.30–7.50%] vs. 9.00% [95% CI 5.20–14.0%], *P* = 0.017) were significantly higher in the emphysema group than those in the normal lung group (Fig. 2). The details of other causes of death were compared between the normal lung and emphysema groups, but no significant differences were found (Supplementary Table 4). In addition, we divided the emphysema group into the mild emphysematous lung group (mild group, 73 cases) and the moderate to severe emphysematous lung group (moderate + severe group, 124 cases) according to the Goddard score and compared the three groups (normal lung vs. mild vs. moderate + severe). Not only the moderate + severe group but also the mild group had higher cumulative incidences of other causes of death (3.50% [95% CI 2.10–5.50%] vs. 16.2% [95% CI 6.60–29.5%] vs. 13.3% [95% CI 7.50–20.8%], *P* < 0.001) and lung cancer-related deaths (5.10% [95% CI 3.30–7.50%] vs. 13.2% [95% CI 5.40–24.6%] vs. 6.80% [95% CI 3.20–12.4%], *P* = 0.012) compared with the normal lung group (Fig. 3).

## Discussion

In the present study, patients with early-stage NSCLC who underwent complete surgical resection were divided into two groups: the normal lung group and the emphysema group. Their clinicopathological backgrounds and prognoses were compared. In addition, the degree of emphysema was evaluated based on the Goddard score, and prognoses were compared according to the severity. Both OS and RFS were significantly poorer in the emphysema group, and highly malignant lung cancer occurred more frequently in the emphysema group than in the normal lung group, as previously reported.^23^ In other words, a higher SUVmax in tumors and higher-grade subtypes of adenocarcinoma were observed in the emphysema group, and for pathological malignant findings, more LY, V, and PL were observed, despite having the same clinical stage between the emphysema and normal lung groups. These results may be attributed to chronic inflammation or the enhanced turnover of alveolar wall cells in emphysematous lungs.^24,25^ The poor prognosis in the emphysema group was suggested to be the result of the high-grade malignancy of the lung cancer that originated in the emphysematous lung.

In contrast, postoperative respiratory complications were more frequent in the emphysema group. Univariable and multivariable analyses of OS revealed that emphysema itself was an independent prognostic factor. The finding that an emphysematous background remained a prognostic factor in multivariable analysis in addition to an SUVmax ≥ 1.8 as variables reflecting tumor malignancy suggest that both the development of high-grade tumors and emphysema itself may contribute to OS. In the multivariable analysis, we included preoperative findings and excluded operative or pathological/postoperative findings, because we attempted to disclose the role of emphysema in prognosis in the preoperative setting to determine a surgical strategy. Pathological findings were included as variables for univariable and multivariable analyses instead of CT findings; however, emphysema remained an independent poor prognostic factor. Propensity score matching also was performed to minimize selection bias, but emphysema remained a significant prognostic factor. These findings suggest that both emphysema and pathological malignancy may affect prognosis.

To explore why emphysema itself remained an independent poor prognostic factor, additional analyses were conducted on the risk of death from other diseases. The analyses revealed that significantly more other causes of death were detected in the emphysema group than in the normal lung group, regardless of the severity of emphysema. In other words, the presence of emphysema, even if mild, tended to result in more deaths than that in the normal lung group. Thus, the risk of other causes of death may be increased in cases of mild emphysema as well as severe emphysema, and this should be considered when deciding on treatment, specifically for aggressively selecting sublobar resection.

In terms of postoperative complications, the finding that respiratory-related complications occurred more frequently in patients with emphysema is reasonable because emphysema is a representative lung disorder that results from damage to the walls of the alveoli in the lungs. Because emphysematous lungs consist of enlarged alveoli with a fragile wall and chronic inflammation, pneumonia and pulmonary fistula are more common in patients with emphysema than in normal lungs after surgical resection.^26^

This study had some limitations. First, this was a retrospective study conducted at a single institution, which could have caused a selection bias. Second, patients with IP were excluded from the analysis. This patient group was excluded, because the prognosis is very poor in patients with IP, which was assumed to interfere with the evaluation of the prognostic impact of emphysema because emphysema and IP are frequently simultaneously present in individual patients.^7,8,27^ Third, respiratory complications were examined in detail; however, other details were not examined. A more detailed examination of patient comorbidities and noncancer-related deaths may help to identify new prognostic factors.

## Conclusions

In patients with early-stage NSCLC, emphysema was indicated to contribute to poor prognosis after complete pulmonary resection, not only by the development of highly malignant tumors but also by increasing the risk of other causes of death that could be attributed to emphysema. Although the results of this study need to be validated in larger cohorts, for cases with emphysema, even if mild, both the high-grade malignancy of lung cancer and the risk of other causes of death should be considered to determine appropriate treatment strategies.

### Supplementary Information

Below is the link to the electronic supplementary material.

Supplementary file1 (DOCX 15 kb)

Supplementary file2 (TIFF 14840 kb)
