# Supplementary material for: The Role of Emphysema on Postoperative Prognosis in Early-Stage Nonsmall Cell Lung Cancer
Source: Ann Surg Oncol. 2024 May 13;31(8):5055–63. doi: 10.1245/s10434-024-15126-x (PMC11236929; doi:10.1245/s10434-024-15126-x)
Supplement: Supplementary file 1 — Supplementary file1 (DOCX 15 kb) [file 10434_2024_15126_MOESM1_ESM.docx]

| Supplementary Table 1. The subtypes of adenocarcinoma | | | |
| --- | --- | --- | --- |
| Grade | Normal lung (n=478) | Emphysema lung (n=126) | *P* |
| Low grade | 178 (37.2%) | 29 (23.0%) | .0019* |
| Moderate grade | 274 (57.3%) | 81 (64.3%) |  |
| High grade | 23 (4.80%) | 14 (11.1%) |  |
| Others | 3 (0.60%) | 2 (1.6%) |  |
| The subtypes of adenocarcinoma are classified as follows: adenocarcinoma in situ, minimally invasive adenocarcinoma, and lepidic adenocarcinoma as low grade; papillary adenocarcinoma and acinar adenocarcinoma as moderate grade; solid adenocarcinoma and micropapillary adenocarcinoma as high grade; and invasive mucinous adenocarcinoma and others as others. | | | |
|  |  |  |  |
|  |  |  |  |
|  |  |  |  |

| Supplementary Table 2. Univariable and Multivariable Cox regression analyses for overall survival using clinical and pathological variables | | | | | |  |
| --- | --- | --- | --- | --- | --- | --- |
|  |  | **Univariable analysis** | | **Multivariable analysis** | |  |
| **Variables** | **Status** | **HR (95% CI)** | ***P*** | **HR (95% CI)** | ***P*** |  |
| Emphysema | Yes *vs.* No | 3.05 (2.00 - 4.64) | <.001* | 2.07 (1.27 - 3.35) | .0031* |  |
| Age (y) | ≥ 70 *vs.* < 70 | 3.40 (2.16 - 5.34) | <.001* | 3.91 (2.45 - 6.26) | <.001* |  |
| Sex | Male *vs.* Female | 2.28 (1.42 - 3.67) | <.001* | 1.64 (0.95 - 2.85) | .077 |  |
| pathological malignant | Yes *vs.* No | 4.02 (2.59 - 6.23) | <.001* | 2.50 (1.53 - 4.08) | <.001* |  |
| pathological size (mm) | > 20 *vs.* ≤ 20 | 1.34 (0.88 - 2.04) | .17 | 1.06 (0.68 - 1.64) | .81 |  |
| SUVmax | > 1.8 *vs.* ≤ 1.8 | 3.08 (1.94 - 4.87) | <.001* | 1.88 (1.12 - 3.18) | .018* |  |
| CCI | ≥ 3 vs. < 3 | 3.63 (2.35 - 5.60) | <.001* | 1.18 (0.62 - 2.25) | .62 |  |
| HR, hazard ratio; CI, confidence interval; SUVmax, maximum standardized uptake value; CCI, Charlson Comorbidity Index. | | | | | |  |
|  |  |  |  |  |  |  |

| Supplementary Table 3. Univariable and Multivariable Cox regression analyses for overall survival using propensity score | | | | | |  |
| --- | --- | --- | --- | --- | --- | --- |
|  |  | **Univariable analysis** | | **Multivariable analysis** | |  |
| **Variables** | **Status** | **HR (95% CI)** | ***P*** | **HR (95% CI)** | ***P*** |  |
| Emphysema | Yes *vs.* No | 3.05 (2.00 - 4.64) | <0.001 | 2.13 (1.30 - 3.47) | .0025* |  |
| Propensity Score |  | 18.72 (7.11 - 49.3) | <0.001 | 8.75 (2.90 - 26.4) | <.001* |  |
| HR, hazard ratio; CI, confidence interval. | | | | | |  |
|  |  |  |  |  |  |  |

| Supplementary Table 4. Details of the other causes of death | | | |
| --- | --- | --- | --- |
| All (n=49) | Normal lung (n=23) | Emphysema lung (n=26) | *P* |
| Pulmonary | 10 (43.5%) | 10 (38.5%) | .903 |
| Cardiovascular | 2 (8.70%) | 2 (7.69%) |  |
| Second lung cancer | 1 (4.35%) | 3 (11.5%) |  |
| Others | 10 (43.5%) | 11 (42.3%) |  |

**Supplementary Figure.** The CONSORT diagram in the present study. A total of 836 patients were included who underwent complete resection at Hiroshima University for clinical stage IA NSCLC between April 2007 and December 2018. Patients with the radiological pattern of IP or who received preoperative induction therapy were excluded. The patients were divided into two groups, 524 with normal lungs (normal lung group) and 197 with emphysematous lungs (emphysema group). NSCLC, non-small cell lung cancer; IP, interstitial pneumonia.
