# Supplementary figures and images for: The Role of Emphysema on Postoperative Prognosis in Early-Stage Nonsmall Cell Lung Cancer
Source: Ann Surg Oncol. 2024 May 13;31(8):5055–63. doi: 10.1245/s10434-024-15126-x (PMC11236929; doi:10.1245/s10434-024-15126-x)

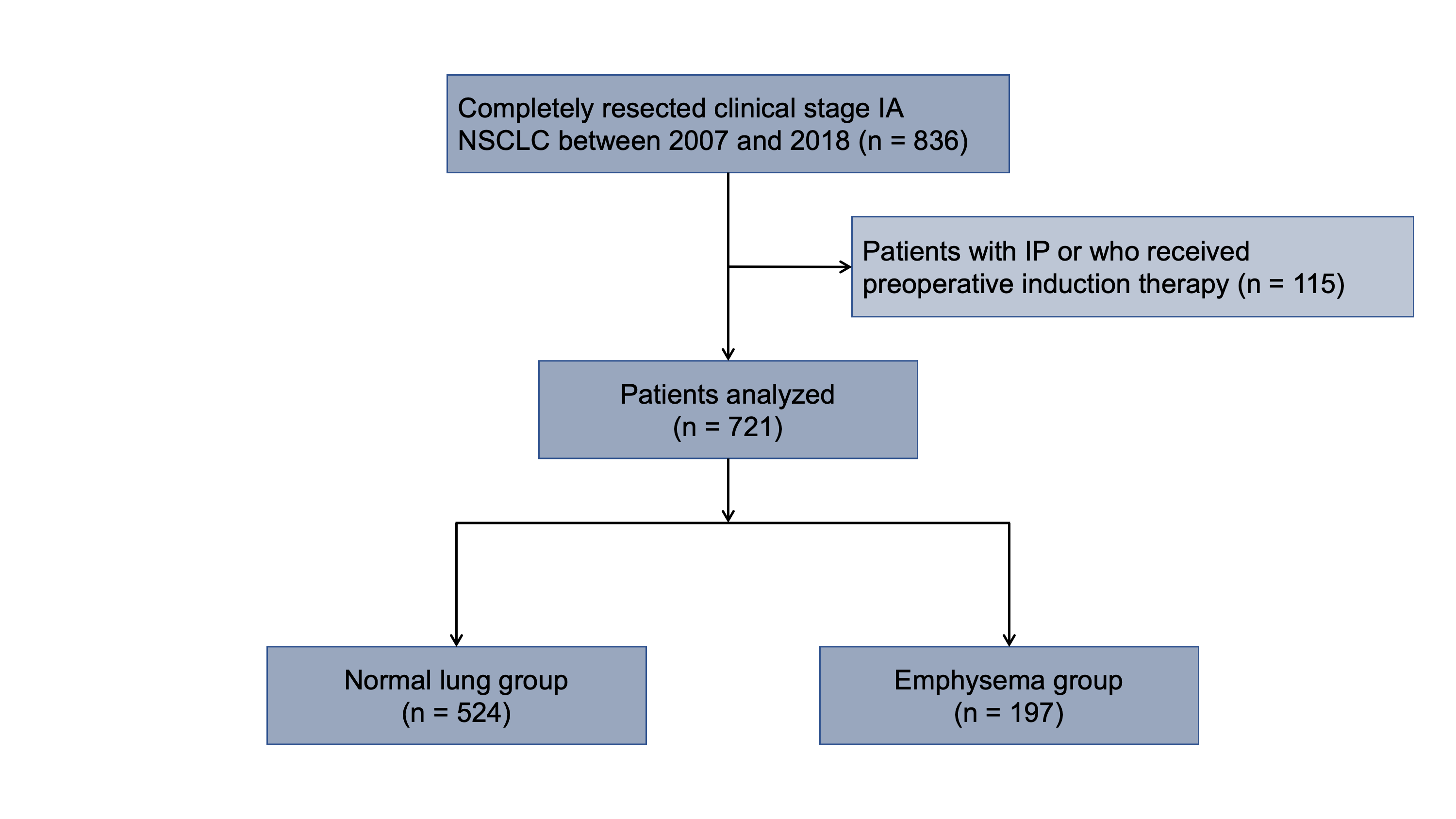

Supplement: Supplementary file 2 — Supplementary file2 (TIFF 14840 kb) [file 10434_2024_15126_MOESM2_ESM.tiff]
